# West Chicago Medical Society

**Published:** 1879-02

**Authors:** J. H. Salisbury


### Article XI.

### West Chicago Medical Society. Report on Chemistry.

Read to the Society by J. H. Salisbury, m. d.

In this paper I shall not attempt to give an account of all the discoveries made during the year, but shall note only those which bear more or less directly on the practice of medicine.

The liquefaction of the gases is one of the most important discoveries made during the past year. The discovery was made almost simultaneously by Cailletet, of Paris, and by Pictet, of Geneva.

The method employed for oxygen is briefly as follows: Oxygen gas is generated in a closed retort until the pressure equals 320 atmospheres. This retort ends in a tube which is surrounded by another tube, in which a temperature of 140° C. is obtained by the evaporation of carbonic dioxide, under greatly diminished pressure.

When the pressure in the retort in which the oxygen is generated has reached 320 atmospheres, a stop-cock is opened and some of the gas is allowed to escape. One portion of the greatly condensed gas in expanding, abstracts sufficient heat from the rest, which is already greatly refrigerated, to reduce it to the liquid condition. The temperature of this liquid has been supposed to be absolute zero.

This discovery seems to confirm the position of hydrogen as a metal. Liquefied hydrogen is described as an opaque bluish liquid, which, in falling on the ground, produces a metalic ring.

Prof. Selmi obtained from the bodies of persons, who had died a natural death, several substances possessing the general characters of the alkaloids, and has found that they are derived from the spontaneous decomposition of the corpses. Some of these substances are fixed, others volatile ; some soluble in ether, others insoluble in that medium, but soluble in amylic alcohol ; others again insoluble in both these liquids. Generally the fixed cadaveric alkaloids yield a precipitate with almost all general re-agents. They yield color re-actions, which, however, are not produced in certain cases, according to the conditions of putrefaction.

These compounds are easily oxidized, and turn brown on exposure to the air, evolving a peculiar urinous odor ; at other times the smell is like that of conia, and sometimes they exhale an odor like that of certain flowers. They generally possess a pungent taste, which benumbs the tongue. Sometimes the flavor is bitter.

Some of these compounds have no effect upon animal life, but others are powerfully poisonous. The symptoms produced by them are transient dilation of the pupils, slackening and irregularity in the pulsations of the heart, and convulsions. After death the heart is found contracted and void of blood.

Prof. Haines, of Chicago, discovered one of these alkaloids previously to Prof. Selmi, and described it in a paper read before the Chicago Chemical Society last winter. He found that it varied somewhat with the degree of decomposition. The alkaloid extracted by Dr. Haines was not poisonous.

It is important to bear in mind the occurrence of these substances, as otherwise an inexperienced analyst might be misled and suppose that he had found poison when he had not.

Some experiments of considerable interest to the medical profession were made by J. J. Coleman, of Glasgow, on "The digestive power of malt liquors."

Finding that during an illness he was greatly benefited by a liquid somewhat like ale, which he calls Hoff's liquid, and which increased his digestive power for starchy food, he determined to examine it.

A certain amount of bread was digested in a given amount of Hoff's liquid, made slightly alkaline for from six to twenty-four hours. A blank experiment was carried on at the same time, in which the same amount of bread was digested in water made slightly alkaline.

Comparative experiments were made at the same time with different specimens of beer, ale, and porter. The average result of the experiments was as follows:

| | | | | | | |
|---|---|---|---|---|---|---|
| Burton ale, dissolved | 15 | per cent. | of the | starch | in the | bread. |
| Wrexam ale, dissolved | 26 | " | " | " | " | " |
| London porter, dissolved | 40 | " | " | " | " | " |
| Hoff's liquid | 60 | " | " | " | " | " |

These experiments show a remarkable solvent power of malt liquors on starch. The author states that the dissolved solids partook more of a gummy than of a saccharine character.

The dissolving or digesting agent is not alcohol; for the stronger ales did not act so powerfully on starch as the weaker ones. Dr. Haines has repeated these experiments with American beers, and found the same digestive power, only in a lower degree. This he attributes to the poorer quality of the beer in America, as according to the admission of some Milwaukee brewers, corn and other grain is substituted for malt to a large extent.

Dyalized iron has been recently used as an antidote for arsenic.

For this purpose it possesses great advantages over ferric hydrate as it can be kept on hand while ferric hydrate must be recently prepared to act with efficiency. Dyalized iron requires to be mixed with some neutral salt before it can act efficiently as an antidote. This neutral salt is ordinarily furnished by the gastric juice, but lest for some reason the gastric juice should fail to furnish this necessary condition, the dyalized iron should be followed by a teaspoonful of common salt, mixed, if possible, with a little calcined magnesia, or other alkaline substance to neutralize the acid of the gastric juice.

A number of cases of arsenical poisoning which have been successfully treated by dyalized iron have been reported, thus confirming the results of experiment.

The presence of arsenic in the preparations of bismuth has excited some attention during the year. In some experiments made by the author and published in the June number of the JOURNAL AND EXAMINER, arsenic was found in thirteen out of eighteen specimens of the sub-nitrate of bismuth, the largest quantity being 1-5 of 1 per cent.

J. W. Underhill, M. D., relates a case, in the *Lancet and Clinic*, in which he gave sub-nitrate of bismuth to his child for eleven days, when the child began to show marked symptoms of arsenical poisoning. The sub-nitrate of bismuth was examined and found to contain 15-100 of 1 per cent. of arsenic. Another specimen showed the presence of 24-100 of 1 per cent.

These facts show that the administration of sub-nitrate of bismuth for a long time and in large doses is dangerous, unless the sub-nitrate is known to be pure.

It is interesting to note the number of organic compounds now made by synthesis from their inorganic elements.

The artificial manufacture of indigo has been made possible during the past year by the discovery of a method of making isatin, which, being deoxidized gives indigo.

The manufacture of artificial alizarin has caused many acres formerly devoted to the culture of madder, to be devoted to other purposes.

Benzoic acid may be successfully made from inorganic sources.

Ether, alcohol, acetic acid, chloroform, hydrate of choral, croton chloral hydrate, urea, nitrous ether and many other compounds, may now be made from their inorganic elements.

These advances point to the time when it may be possible to manufacture cheaply the medicines which are now obtained at great cost from their natural sources.

I will close what I have to say with the following words from an address by Prof. Crooks :

" Most medical men look on chemistry as a kind of adjunct to pharmacy—a knowledge of which will prevent them from poisoning their patients or from puzzling their pharmacists by prescribing a series of incompatibles. To such we would put a simple question : How is it that although the chemist during the last ten years has discovered many thousands of definite compounds possessing the most multifarious chemical, physical, and presumably physiological properties that not half a dozen of these have been applied to the relief of human suffering or the saving of human life? Is it possible, even according to the vulgar doctrine of chance, that there are no compounds lying *perdu* on the shelves of the chemist's laboratory that would prove as powerful weapons in the hands of the physician, as chloroform, chloral, or amyl nitrite?

"The field open to the medical student in this direction is surely vast enough to content the hardest worker, and would surely be fruitful enough to satisfy the highest ambition."

---

Origin of Amniotic Fluid.—Gusserow. (*Arch. f. Gynäk. XIII.*)

In 1871 G. demonstrated that soluble substances would pass by way of the circulation from mother to child, and from the latter to the amniotic fluid. He claims that this fluid is not only an exclusively fœtal product, but that during the later months of pregnancy it is mostly secreted by its kidneys.

Runge and Schmiedeberg proved that the conversion of benzoic into hippuric acid occurs only in the kidney. If hippuric acid be found in the urine of the fœtus after the mother had taken benzoic acid, the former must perforce have been formed in the kidneys of the fœtus, the blood carried to the latter being charged with benzoic acid. So, if hippuric acid be found in the amniotic fluid it must have been formed in the kidneys also, for if that fluid were merely a transudate, benzoic and not hippuric acid would be present.

Gusserow shows in a striking way that both the urine and amniotic fluid of the child contain hippuric acid when one to two grams of sodic benzoate were administered to the parturient several hours prior to rupture of the membranes

From these and such experiments as the injection of strychnia, he is led to believe that the amniotic fluid is mainly an excretion of the child, though it may have other minor sources.

